# KPT‐330 and Y219 exert a synergistic antitumor effect in triple‐negative breast cancer through inhibiting NF‐κB signaling

**DOI:** 10.1002/2211-5463.13588

**Published:** 2023-03-20

**Authors:** Tiantian Wen, Mengzhu Geng, Enhe Bai, Xueyuan Wang, Hang Miao, Zhimeng Chen, Hui Zhou, Jia Wang, Jingmiao Shi, Yin Zhang, Meng Lei, Yongqiang Zhu

**Affiliations:** ^1^ College of Life Science Nanjing Normal University China; ^2^ College of Science Nanjing Forestry University China; ^3^ Jiangsu Chia Tai Fenghai Pharmaceutical Co. Ltd. Nanjing China; ^4^ School of Food Science and Pharmaceutical Engineering Nanjing Normal University China

**Keywords:** KPT‐330, NF‐κB, triple‐negative breast cancer, Y219

## Abstract

Triple‐negative breast cancer (TNBC) is an aggressive breast cancer subtype, which has poor prognosis due to the lack of effective targeted drugs. KPT‐330, an inhibitor of the nuclear export protein CRM‐1, has been widely used in clinical medicine. Y219, a novel proteasome inhibitor designed by our group, shows superior efficacy, reduced toxicity, and reduced off‐target effects as compared to the proteasome inhibitor bortezomib. In this study, we investigated the synergistic effect of KPT‐330 and Y219 against TNBC cells, as well as the underlying mechanisms. We report that combination treatment with KPT‐330 and Y219 synergistically inhibited the viability of TNBC cells *in vitro* and *in vivo*. Further analysis revealed that the combined use of KPT‐330 and Y219 induced G2‐M phase arrest and apoptosis in TNBC cells, and attenuated nuclear factor kappa B (NF‐κB) signaling by facilitating nuclear localization of IκB‐α. Collectively, these results suggest that the combined use of KPT‐330 and Y219 may be an effective therapeutic strategy for the treatment of TNBC.

## Graphical abstract

We investigated the synergistic effect of KPT‐330 and Y219 against TNBC cells, as well as the underlying mechanisms. Combining KPT‐330 and Y219 induced G2‐M phase arrest and apoptosis in TNBC cells, and attenuated NF‐κB signaling by facilitating nuclear localization of IκB‐α. These results suggest that combining KPT‐330 and Y219 may be an effective therapeutic strategy for treating TNBC.

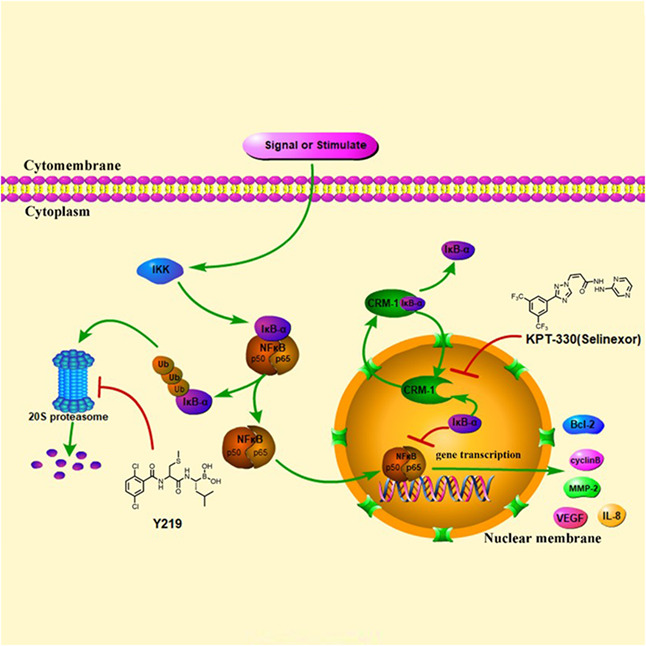

**Abbreviations**
Bcl‐2: B‐cell lymphoma‐2
CCK‐8: cell counting Kit‐8
CDKs: cyclin‐dependent kinases
CI: combination index
CRM‐1: chromosome maintenance protein 1
Cyto‐*c*
: cytochrome *c*
FDA: Food and Drug Administration
I.V.: intravenous
IHC: immunohistochemistry
IκBα: inhibitor of nuclear factor kappa‐B alpha
IL‐8: interleukin‐8
MM: multiple myeloma
MMP‐2: matrix metallo proteinase
NES: nuclear export signal
NF‐κB: nuclear factor κB
NLS: nuclear localization sequence
P.O.: Oral
PIs: proteasome inhibitors
SINE: selective inhibitors of nuclear export
TNBC: triple‐negative breast cancer
TNF‐α: tumor necrosis factor‐α
TUNEL: terminal deoxynueleotidyl transferase‐mediated dUTP nick endlabeling
UPP: ubiquitin‐proteasome pathway
VEGF: vascular endothelial growth factor

Triple‐negative breast cancer (TNBC) accounts for 15–20% of breast cancers, which is characterized as a subclass that estrogen receptor (ER), progesterone receptor (PR), and human epidermal growth factor receptor‐2 (HER‐2) deficient in the plasma membrane [1, 2]. Although classical chemotherapy at an early stage could prolong the survival of patients with TNBC, there is still a lack of effective comprehensive therapeutic regimens due to the high rate of recurrence and distant metastasis of the disease [3, 4]. Previous studies have demonstrated that the activated nuclear factor kappa B (NF‐κB) signaling pathway is associated with poor prognosis in patients with TNBC, yet little is known about the underlying mechanisms [5, 6].

Bortezomib, a kind of proteasome inhibitor (PI), is proven as a potential therapeutic agent for treating TNBC due to its pharmacological control of the NF‐κB signaling pathway [7]. Bortezomib is the first PI approved by the FDA against refractory multiple myeloma (MM) [8, 9, 10], which is covalently bound to the threonine residue of the β5 subunit in the 20S proteasome complex so as to inhibit the ubiquitin‐proteasome pathway (UPP) [9, 10, 11]. In malignant cells, the UPP is involved in regulating tumor cell cycle, apoptosis, and tissue angiogenesis via regulating the NF‐κB signaling pathway [3, 12, 13, 14]. Despite the potent antitumor activity, bortezomib as well as other PIs, such as ixazomib and carfilzomib, have dose‐related toxicities, including neurotoxicity, thrombocytopenia, neutropenia, and cardiotoxicity [15, 16, 17, 18]. Accordingly, we synthesized a promising candidate, Y219, which shows good inhibitory activity against the 20S proteasome and exerts excellent antitumor effects on different MM models [19, 20].

The accurate cellular localization of protein is crucial to maintain cellular metabolism and homeostasis. Nuclear‐cytoplasmic transport is an important cellular event, and defects in this process may lead to uncontrolled cell proliferation and malignant transformation [21]. Chromosomal region maintenance‐l (CRM‐1) is a nuclear output transporter and mediates the transport of multiple proteins, including tumor suppressor proteins (e.g. p53, FOXO, p73) and cell‐cycle regulators (e.g. p21^CIP1/WAF1^, p27^KIP1^, Rb1) [22, 23, 24]. CRM1‐mediated nuclear export is etiologically associated with various types of cancers, including pancreatic cancer, gastric cancer, and osteosarcoma, which has become an important clinical diagnostic indicator and therapeutic target [25, 26, 27, 28]. Nowadays, the most effective oral Selective Inhibitors of Nuclear Export (SINE) focus on targeting the Cys528 residue of NES‐binding groove in CRM‐1 through Michael addition [29], including KPT‐185, KPT‐251, KPT‐276, and KPT‐330 (Selinexor). It has been reported that KPT‐330 shows antitumor effects as well as good safety, tolerability on solid tumors through inhibiting nuclear export of IκB, and the resultant inactivation of NF‐κB signaling [27, 30, 31, 32, 33].

PI‐SINE combined administration is considered a promising strategy on blood tumors (e.g. MM) and solid tumors (e.g. colon cancer). In this study, we evaluated the pharmacodynamics of Y219‐KPT‐330 combined administration against TNBC *in vitro* and *in vivo*, and investigated the potential mechanism of their synergistic effect.

## Materials and methods

### Cell lines

The breast cancer cell lines MDA‐MB‐231, HCC‐1937, and 4T1 were purchased from the Type Culture Collection of the Chinese Academy of Sciences (Shanghai, China). Breast cancer cell lines were maintained in RPMI‐1640 medium supplemented with 10% (v/v) fetal bovine serum (FBS; Gibco) and 1% (v/v) penicillin/streptomycin (Gibco) at 37 °C with 5% CO_2_. The human normal breast MCF‐10A cell line was offered by Prof. Zhigang Hu (College of Life Science, Nanjing Normal University) and maintained in Dulbecco's modified Eagle medium (DMEM) with 10% FBS (Gibco, Thornton, NSW, Australia) at 37 °C with 5% CO_2_.

### Cell viability analysis

Cells (HCC‐1937, 4T1, and MCF‐10A) were seeded in 96‐well plates at 2000 cells per well and incubated in the presence of Y219 (0–100 nm) and KPT‐330 (2–1000 nm) at 37 °C for 72 h. MDA‐MB‐231 cells incubated in the presence of Y219 (0–100 nm) and KPT‐330 (0–500 nm) for 72 h. The cell viability assay was determined using a cell proliferation assay with the Cell Counting Kit‐8 (CCK‐8) following the manufacturer's specifications (Dojindo Laboratories, Kumamoto, Japan).

### Evaluation of drug combination index (CI)

Cells (HCC‐1937, 4T1, and MCF‐10A) were seeded in 96‐well plates and incubated in the presence of Y219 (1–6 nm) and KPT‐330 (2–1000 nm) at 37 °C for 48 h. MDA‐MB‐231 cells incubated in the presence of Y219 (1–6 nm) and KPT‐330 (0–500 nm) for 48 h. Cell viability was measured using the CCK‐8 assay. The drug CI values of drugs were calculated as previously described [34].

### Cell cycle and apoptosis assays

Cells were incubated with KPT‐330 (100 nm) and/or Y219 (6 nm) for 48 h. Cellular DNA content was quantified using PI/RNase Staining Buffer (BD Biosciences, San Diego, CA, USA). Cell apoptosis was detected using the Annexin V/PI staining assay kit (BD Biosciences). Cell cycle and apoptosis were analyzed by flow cytometry.

### *In vivo* activity of KPT‐330 and Y219 in MDA‐MB‐231 xenograft models

All animal studies described here were performed according to protocols approved by Institutional Animal Care and Use Committee of Nanjing Normal University (IACUC‐20200506). Six‐week‐old female BALB/ nude mice (Shanghai SIPPR‐BK Laboratory Animal Co. Ltd., Shanghai, China) were housed in well‐ventilated cages with access to standard diet and water *ad libitum* under a 12‐h light–dark cycle and constant ambient temperature. To establish the MDA‐MB‐231 xenograft model, 1 × 10^6^ MDA‐MB‐231 cells were suspended in 50% Matrigel matrix (Becton Dickinson, San Jose, CA, USA) and injected subcutaneously in the right forelimb of mice. When the mean tumor volume reached 80 mm^3^, the mice were randomized into a vehicle group (p.o.: 150 μL per mouse; twice a week for 3 weeks; *n* = 5), a Y219 group (i.v.: 0.4 mg·kg^−1^; twice a week for 3 weeks; *n* = 5), a KPT‐330 group (p.o.: 12.5 mg·kg^−1^; once 1 days for 3 weeks; *n* = 5), and a KPT‐330 + Y219 combination group (*n* = 5). The body weight and tumor volume of the mice were recorded every other day. The tumor volume was calculated using the following formula: 0.5 × length × width^2^. All mice survived the experiment and were euthanized by CO_2_ asphyxiation and all the tumor tissues were harvested, collected, weighed, and photographed.

### Immunohistochemistry (IHC) staining

The harvested tumor tissues were fixed in 4% formalin, embedded in paraffin, and cut into sections. Then IHC staining was performed using the EnVision Kit (Dako, Glostrup, Denmark) according to the manufacturer's directions. The histologic images were captured using an optical microscope and analyzed with imagej software (National Institutes of Health，Bethesda, Maryland, USA).

### Terminal deoxynucleotidyl transferase dUTP nick‐end labeling (TUNEL) assay

Apoptosis of tumor cells in the xenografts was detected with the TUNEL technique, which assesses DNA fragmentation of apoptotic cells. The microscopic images were captured using an optical microscope and analyzed the area of the positive cells in micrographed with imagej software.

### Western blot analysis

Cytoplasmic and nuclear protein extracts were obtained by using the Nuclear/Cytosol Protein Extraction Kit from Beyotime Biotechnology (Shanghai, China). Proteins were separated by sodium dodecyl sulfate‐polyacrylamide gel electrophoresis (SDS/PAGE) and transferred onto polyvinylidene difluoride (PVDF) membranes. Membranes were blocked with 5% skim milk in Tris‐buffered saline containing 0.1% Tween‐20 (TBST). Membranes were then incubated with primary antibodies at 4 °C overnight, followed by incubation with a peroxidase‐conjugated secondary antibody at room temperature for 1.5 h. Protein bands were visualized using chemiluminescence imaging reagents (ECL; Servicebio, Wuhan, Hubei, China). Primary antibodies: Caspase‐3 (9662), Caspase‐8 (4927), Caspase‐9 (9502), RAPR (9532), Cyto‐*c* (11940), IκB‐α (9242), β‐actin (4970), p65 (8242S), Lamin B1 (13435S), Lamin A/C (4777T), Tubulin (2148S), and Brg1 (49360T) were purchased from Cell Signaling Technology (Shanghai, China). And p53 (sc‐47,698), p21^CIP1/WAF1^ (sc‐24,559), p27^KIP1^ (sc‐1641), CCNB1 (sc‐70,898), Bcl‐2 (sc‐7382), Bax (sc‐7480), CRM1 (sc‐74,454), and MMP‐2 (sc‐13,595) were purchased from Santa Cruz Biotechnology (Inc., Texas, CA, USA). IL‐8 (BS3479), VEGF (AP0742), GAPDH (AP0063), and Survivin (BS1771) were purchased from Bioworld Technology, Inc. (Nanjing, Jiangsu, China).

### NF‐κB activity assay

To determine the inhibition of NF‐κB signaling, the transfected NF‐κB/luciferase 293T cells were pretreated with KPT‐330 (100 nm) and/or Y219 (6 nm) for 6 h and then stimulated with 10 ng·mL^−1^ of tumor necrosis factor‐α (TNF‐α) for 18 h, followed by measurement of luciferase activity by using the Dual‐Lumi Luciferase Reporter Gene Assay Kit (Beyotime). Firefly luciferase activity was measured and normalized to Renilla luciferase activity.

### Quantitative real‐time polymerase chain reaction (RT‐PCR)

Total RNA was extracted using TRIzol reagent (Invitrogen; Thermo Fisher Scientific, Inc., Waltham, MA, USA). Real‐time qPCR was performed using the HiScript III RT SuperMix for qPCR (Vazyme) and ChamQ Universal SYBR qPCR Master Mix (Vazyme, Nanjing, Jiangsu, China) according to the manufacturer's protocols (forward and reverse primer; Table S1). The relative expressions of all genes were quantified using the $2^{-\Delta \Delta C_{T}}$ method. β‐actin was used as the internal control.

### Statistical analysis

Values were expressed as the mean ± standard deviation (SD) of three independent experiments. The two‐tailed Student's *t*‐test was used for comparisons of two groups. Statistical significance was defined as *P* < 0.05. Statistical analysis was performed using graphpad prism 5 (GraphPad Software, Inc., San Diego, CA, USA).

## Results

### KPT‐330 and Y219 synergistically inhibited the cell viability of TNBC cells

**Fig. 1:**
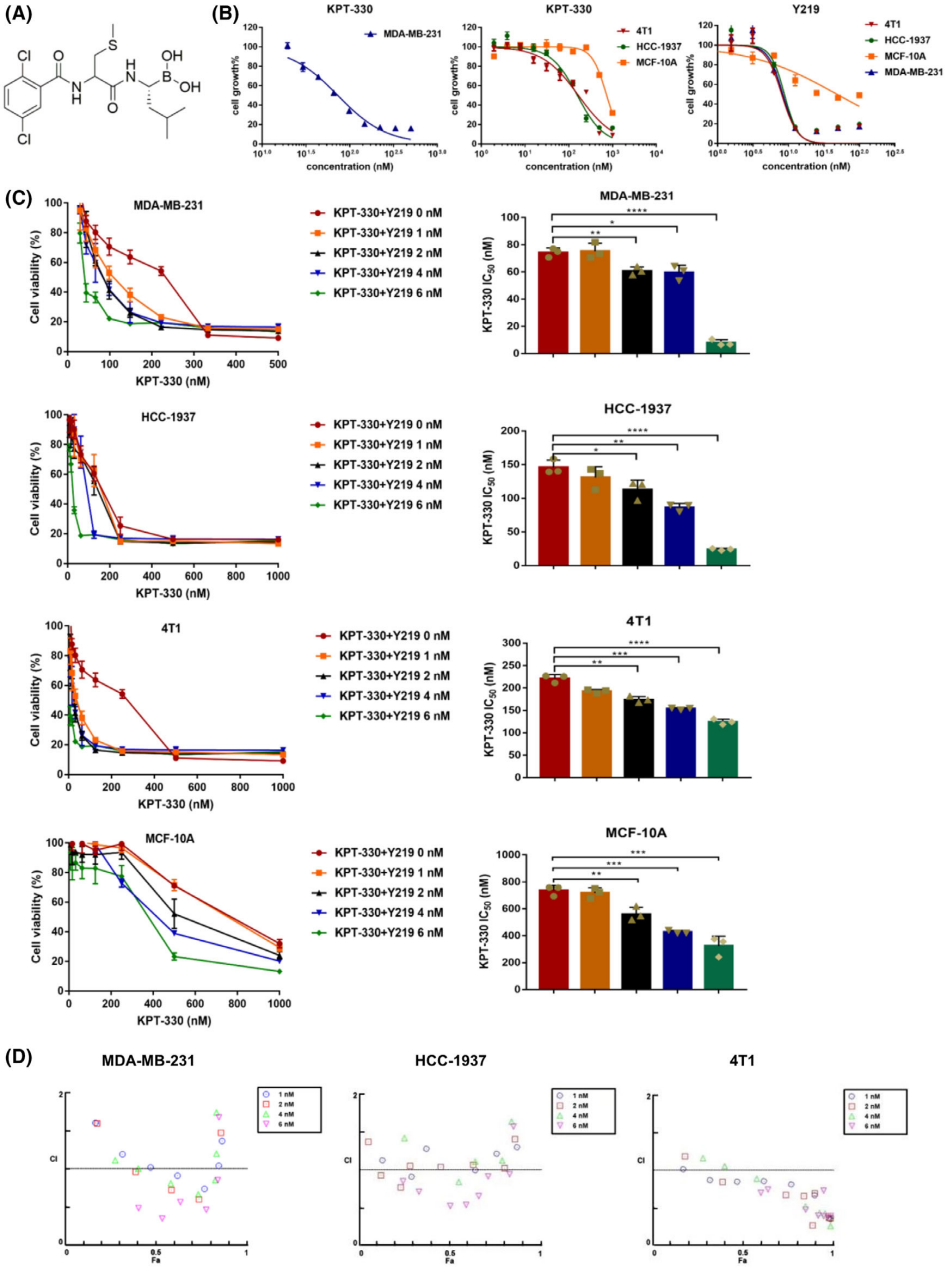
KPT‐330 and Y219 synergistically inhibited the cell viability in TNBC cells. (A) The chemical structure of proteasome inhibitor Y219. (B) The cytotoxic effects of KPT‐330 and Y219 were measured by the CCK‐8 assay in different cell lines. (C) The combined therapy decreased IC_50_ of KPT‐330. Data are presented as mean ± SD (*n* = 3), one‐way ANOVA was used for statistical analysis, *P < 0.05, **P < 0.01, ***P < 0.001, ****P < 0.0001. (D) Combination therapy resulted in a better synergistic inhibitory effect. Antagonism effect (CI > 1), synergistic effect (CI < 1), and additive effect (CI = 1).

Y219, a novel proteasome inhibitor, was chemically synthesized by our group and the structure is shown in Fig. 1A. The CCK‐8 assay was performed to evaluate the effect of KPT‐330 and Y219 on the viability of TNBC cells, including MDA‐MB‐231, 4T1, and HCC‐1937. As shown in Fig. 1B and Table S2, the half‐maximal inhibitory concentrations (IC_50_) values were in the range of approximately from 80 to 230 nm for KPT‐330, while in the range of approximately 20 to 35 nm for Y219 in TNBC cell lines, indicating that Y219 exerts higher cytotoxicity than KPT‐330. In addition, the IC_50_ values were 736.90 ± 30.76 nm for KPT‐330, while 40.21 ± 6.69 nm for Y219 in normal breast MCF‐10A cells. To investigate the synergistic inhibitory effects of KPT‐330 and Y219 on cell viability, TNBC cells were treated with gradient concentrations of Y219 (1–6 nm) and KPT‐330 (0–500 nm or 2–1000 nm). Compared with KPT‐330 alone, the combined treatment of KPT‐330 and Y219 gradually reduced the IC_50_ values and enhanced the inhibitory effects on cell viability, which reached the lowest value when the concentration of Y219 was 6 nm (Fig. 1C). Notably, the combination of KPT‐330 (100 nm) and Y219 (6 nm) did not significantly affect the viability of the normal breast MCF‐10A cell line, which retained more than 80% viability. In addition, the CI analysis revealed that the synergistic inhibitory effects were observed in MDA‐MB‐231 and HCC‐1937 cells, but not in 4T1 cells (Fig. 1D and Table S3). It is worth pointing out that the optimal concentrations of KPT‐330 and Y219 were 100 and 6 nm, respectively, which exhibited a better synergistic effect, and was used as a dosing regimen for subsequent *in vitro* experiments.

### Combination of KPT‐330 and Y219 triggers G2‐M cell cycle arrest and apoptosis

**Fig. 2:**
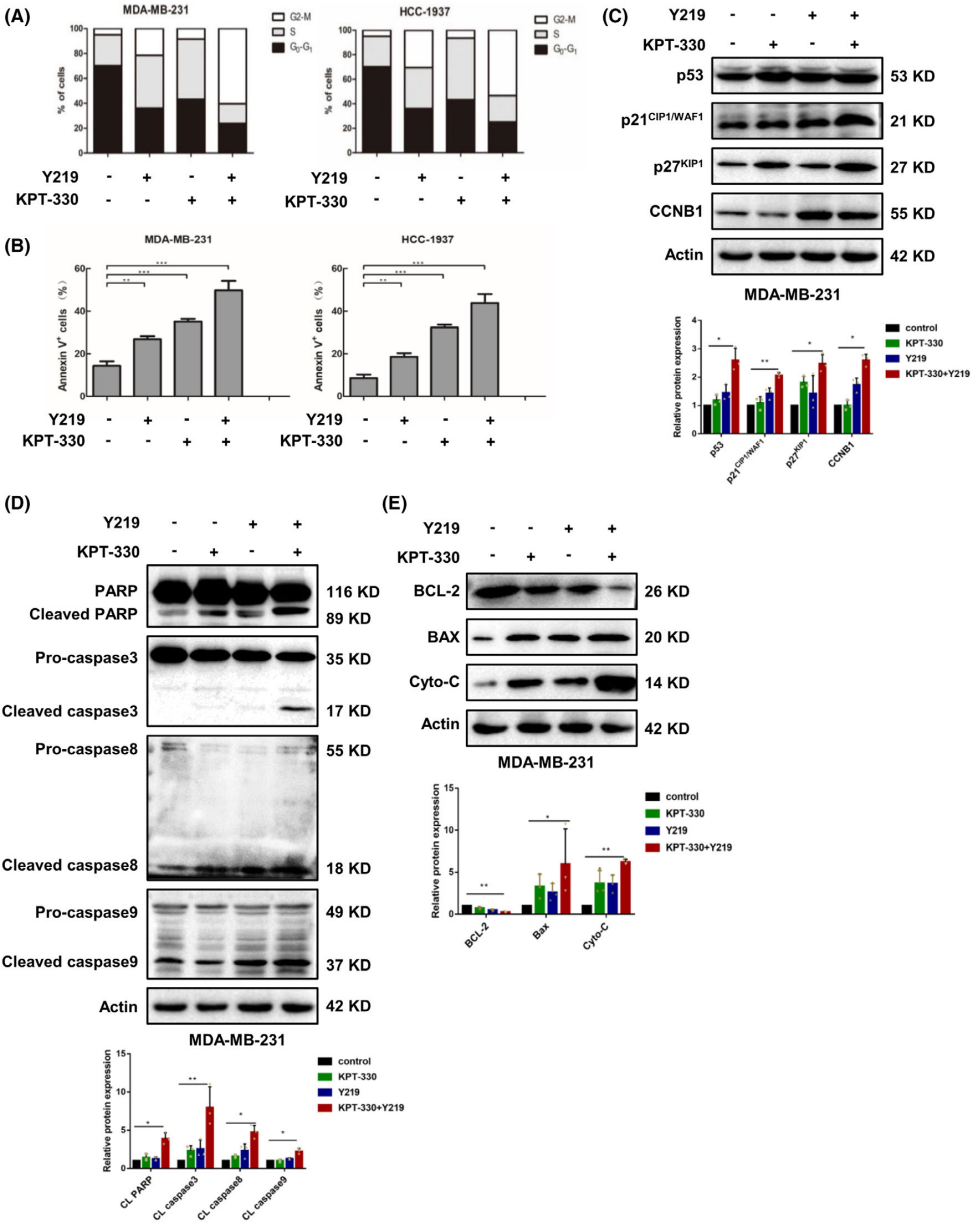
Combination of KPT‐330 and Y219 triggers G2‐M cell cycle arrest and apoptosis. (A) Cells were incubated with KPT‐330 (100 nm) and/or Y219 (6 nm) for 48 h.. Cell cycle was analyzed by flow cytometry. (B) Induction of apoptosis was determined with Annexin V^+^/PI^−^ staining by flow cytometry. (C–E) The protein expression levels of cell‐cycle‐related proteins (p53, p21^CIP1/WAF1^, p27^KIP1^, and CCNB1) and apoptosis‐related proteins (cleaved caspase 3/8/9, PARP, BCL‐2, BAX, and Cyto‐*c*) were determined by western blot in MDA‐MB‐231 cells treated with KPT‐330 (100 nm) and/or Y219 (6 nm) for 48 h. Actin was used as a control. Data are presented as mean ± SD (*n* = 3), one‐way ANOVA was used for statistical analysis, *P < 0.05, **P < 0.01, ***P < 0.001.

Next, the effect of the combination of KPT‐330 and Y219 on cell cycle distribution in MDA‐MB‐231 and HCC‐1937 cells was investigated. Compared with Y219 or KPT‐330 alone, the percentage of cells treated with KPT‐330 and Y219 at G0‐G1 and S phases decreased significantly, whereas that of cells at the G2‐M phase was increased (Fig. 2A). Apoptosis commonly is associated with cell cycle arrest. Therefore, we explored the occurrence of apoptosis triggered by KPT‐330 and Y219 in these cells. Apoptotic cells were evaluated by Annexin V‐FITC/PI (Annexin V^+^/PI^−^) staining and flow cytometry analysis. The combination treatment significantly increased the apoptosis rate compared with KPT‐330 or Y219 alone in the MDA‐MB‐231 and HCC‐1937 cells (Fig. 2B).

Encouraged by the above results, we further detected the expression levels of cell‐cycle‐related proteins, which function as tumor suppressors, in MDA‐MB‐231 cells by western blot, including p53, p21^CIP1/WAF1^, p27^KIP1^, and cyclin B1 (CCNB1). Compared with KPT‐330 or Y219 alone, the protein expression levels of these cycle‐related proteins were obviously elevated in MDA‐MB‐231 cells treated with KPT‐330 and Y219 (Fig. 2C and Fig. S1A). Furthermore, combinational treatment markedly induced the cleavage of apoptosis‐related proteins, such as caspase 3, 8, 9, and PARP compared with a single treatment (Fig. 2D and Fig. S1B). The antiapoptotic protein BCL‐2 was dramatically downregulated, while proapoptotic proteins BAX and Cytochrome *c* (Cyto‐*c*) were upregulated (Fig. 2E and Fig. S1C). Taken together, the results indicated that a combination of KPT‐330 and Y219 exerted synergistic effects by induction of G2‐M cell cycle arrest and apoptosis in TNBC cells.

### KPT‐330 and Y219 synergistically inhibit NF‐κB activity

**Fig. 3:**
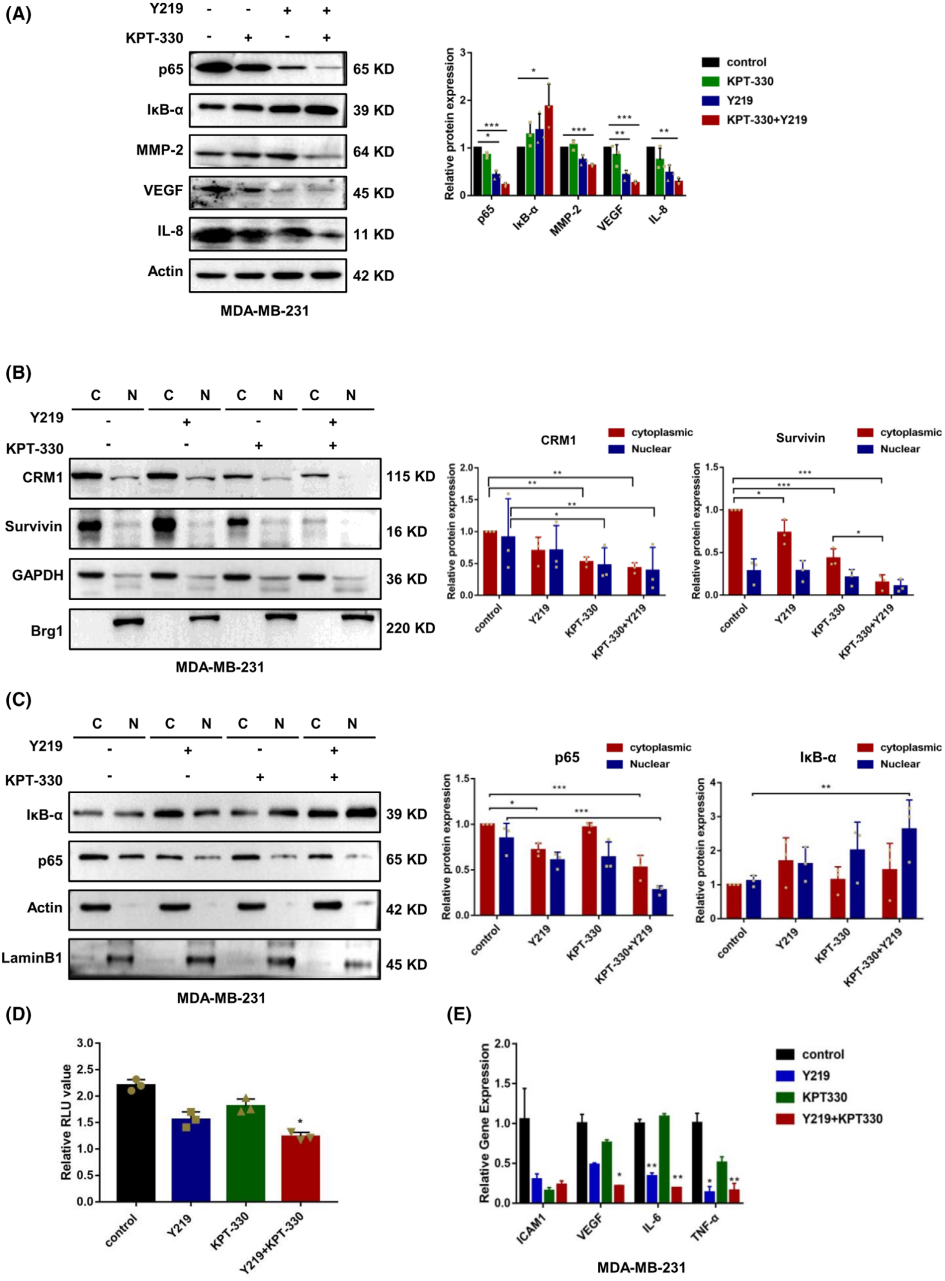
KPT‐330 and Y219 synergistically inhibits NF‐κB activity. (A) The protein levels of NF‐κB‐related proteins (p65, IκB‐α, MMP‐2, VEGF, and IL‐8) were detected by western blot in MDA‐MB‐231 cells treated with Y219 (6 nm) and/or KPT‐330 (100 nm) for 48 h. Actin was used as a control. (B, C) Nuclear and cytoplasmic proteins of CRM1, Survivin, p65, and IκB‐α were determined by western blot in MDA‐MB‐231 cells treated with Y219 (6 nm) and/or KPT‐330 (100 nm) for 48 h. GAPDH, Actin, Brg1, and Lamin B1 served as the internal controls. (D) The transcriptional activity of NF‐κB was determined by using the Dual‐Luciferase Reporter Assay System. (E) The mRNA expression levels of ICAM1, TNF‐α, IL‐6, and VEGF in MDA‐MB‐231 cells were analyzed by Q‐PCR. Data are presented as mean ± SD (*n* = 3), one‐way ANOVA was used for statistical analysis, *P < 0.05, **P < 0.01, ***P < 0.001.

Considering that NF‐κB modulates the expression of various genes essential for cell survival and cell growth, we therefore investigated whether the combination treatment affects the NF‐κB activity in MDA‐MB‐231 cells. Through western blot analysis, we observed that the combination treatment dramatically inhibited the expression of p65, which is the main executor of transcriptional activity in the NF‐κB signaling pathway (Fig. 3A and Fig. S2A). Expectedly, NF‐κB related proinflammatory cytokine IL‐8, growth factor VEGF, and endogenous matrix metalloproteinase MMP‐2 were downregulated, whereas IκB‐α, an inhibitor of NF‐κB, was elevated in MDA‐MB‐231 cells. We then explored the nuclear and cytoplasmic proteins expression levels of CRM1, Survivin, p65, and IκB‐α in MDA‐MB‐231 cells. The results showed that nuclear and cytoplasmic expression levels of CRM1 and p65 were reduced in MDA‐MB‐231 cells treated with KPT‐330 or KPT‐330 + Y219. However, Y219 had no significant effect on the nuclear and cytoplasmic distribution of CRM1 proteins in MDA‐MB‐231 cells (Fig. 3B,C and Fig. S2B,C). In addition, the combination treatment inhibited the cytoplasmic proteins of Survivin in MDA‐MB‐231, but there was no significant effect on the nuclear distribution of Survivin. More important, the combination treatment facilitated the accumulation of IκB‐α in the nucleus of MDA‐MB‐231 cells (Fig. 3C and Fig. S2C), indicating that the combination therapy inhibits the NF‐κB signaling pathway via regulating IκB‐α nuclear localization.

Therefore, as a next step we examined whether Y219 + KPT‐330 could reduce NF‐κB activity. The 293T cells were transfected with luciferase reporter vector under the control of the NF‐κB‐responsive element. NF‐κB was rapidly activated by stimulation with TNF‐α, inducing an increase in the luciferase activity. The results showed that pretreatment of NF‐κB/Luc 293T cells with Y219 + KPT‐330 led to a significant reduction of luciferase activity, and inhibiting the mRNA expression of downstream target genes of NF‐κB, including ICAM1, TNF‐α, IL‐6, and VEGF (Fig. 3D,E).

### Combination of KPT‐330 and Y219 attenuates MDA‐MB‐231 xenograft growth *in vivo*

**Fig. 4:**
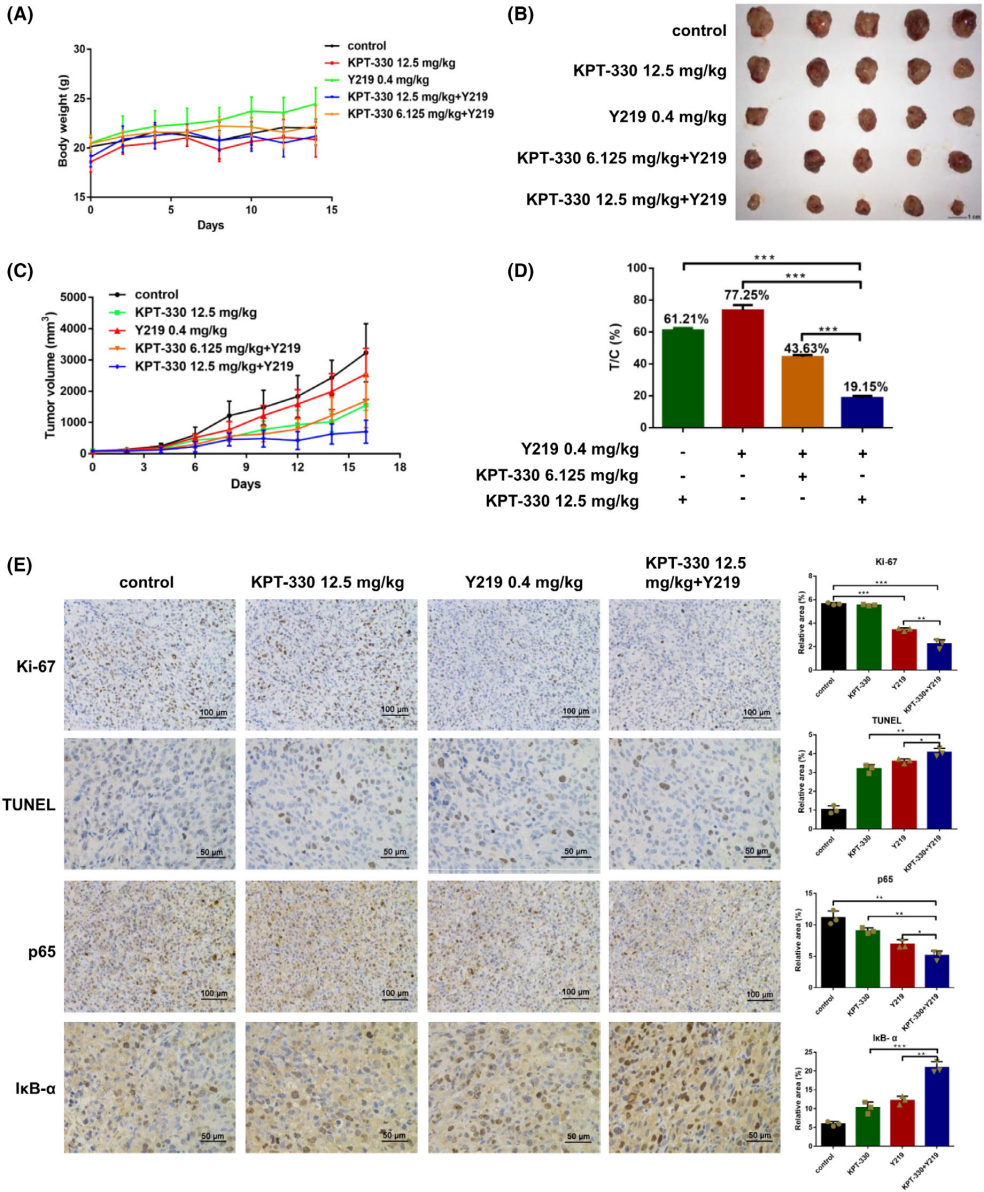
Combination of KPT‐330 and Y219 attenuates MDA‐MB‐231 xenograft growth *in vivo*. (A) Average weight of mice in the vehicle and treatment groups. (B) Differences in tumor size for the vehicle and treatment groups. (C) The measurement of tumor was performed using a caliper. (D) The relative growth rate (T/C%) of xenograft tumors. Data are shown as mean ± SD (*n* = 5), one‐way ANOVA was used for statistical analysis, ***P < 0.001. (E) Protein expression of Ki‐67, p65, and IκB‐α was detected by IHC staining, and apoptosis of tumor cells in the xenografts was analyzed by the TUNEL technique (Scale bar, 50 μm). Data are presented as mean ± SD (*n* = 3), one‐way ANOVA was used for statistical analysis, *P < 0.05, **P < 0.01, ***P < 0.001.

The antitumor efficacy was further evaluated using MDA‐MB‐231 xenograft mouse models. Compared with KPT‐330 or Y219 alone, the combination therapy of KPT‐330 (high‐dose group: 12.5 mg·kg^−1^ or low‐dose group: 6.125 mg·kg^−1^) and Y219 (0.4 mg·kg^−1^) significantly attenuated the growth of MDA‐MB‐231 xenografts, without affecting the body weights at the endpoint of the experiment (Fig. 4A–C). The relative growth rates of single‐agent therapy were 61.21% and 77.25% for KPT‐330 and Y219, respectively. The combination therapy profoundly reduced the relative growth rates (T/C%) of xenograft tumors (low‐dose group: 43.63% and high‐dose group: 19.15%), compared with the single‐agent KPT‐330 (61.21%) or Y219 (77.25%) treatment (Fig. 4D).

Subsequently, xenograft tumors were subjected to IHC staining. The protein expression of Ki‐67 was not significantly downregulated in KPT‐330‐treated xenografts. But the combination of a high‐dose of KPT‐330 (12.5 mg·kg^−1^) and Y219 (0.4 mg·kg^−1^) resulted in downregulation of Ki‐67 compared with control or Y219 administration (Fig. 4E). Moreover, the population of TUNEL‐positive cells in xenografts received from combination therapy were increased, indicating that the combination exerted a proapoptotic effect. Consistent with the *in vitro* results, p65 was significantly decreased in xenografts after combination treatment, while IκB‐α was markedly increased (Fig. 4E).

## Discussion

Previous studies on proteasome inhibitors (PIs) have demonstrated that the suppression of NF‐κB activity was achieved by mitigating the ubiquitination degradation of IκB‐α. Furthermore, nuclear export protein inhibitors, such as ratjadones, were covalently bound to CRM‐1, thus blocking the CRM1‐mediated nuclear export of proteins, including IκB‐α [35]. Our group has previously reported that Y219 exhibited potential antitumor activity in both MM cell lines and primary samples *in vitro* [20]. In this study, we showed that the combination treatment of KPT‐330 and Y219 synergistically inhibited the cell viability of TNBC cells *in vitro* and *in vivo*.

The flow cytometry results showed that the combination therapy induced cell cycle arrest at the G2‐M phase, and ultimately resulted in apoptosis in both MDA‐MB‐231 and HCC‐1937 cells. Western blot analysis revealed that the combination therapy induced the expression levels of p53, p21^CIP1/WAF1^, p27^KIP1^, and CCNB1 in MDA‐MB‐231 cells, which were endogenous cyclin‐dependent protein kinase inhibitors (CDKs) or negative regulators of cell cycle progression [36]. Since the degradation of these proteins was dependent on the UPP pathway, it was speculated that Y219 inhibited proteasome activity and resulted in an accumulation of these regulators, which induced cell cycle arrest in early mitosis and induction of caspase‐dependent apoptosis. Furthermore, the combination therapy triggered apoptosis by activation of the caspase cascades, including caspase‐3, 8, 9, and PARP cleavage. Bcl‐2 is well known to have antiapoptotic activities and confers cellular resistance to a wide range of chemotherapeutic drugs. In this study we observed that the combination therapy decreased the expression of Bcl‐2, thus affecting the release of Cyto‐*c*, which is necessary for the induction of apoptosis.

NF‐κB binds to DNA and initiates transcription of multiple genes to facilitate cancer development and progression, including antiapoptosis and proliferation‐related genes [37]. In cytoplasm, NF‐κB is a heterodimer of the p65 and p50 subunits, and forms a ternary complex with IκB‐α. Phosphorylation of IκB‐α is mediated by IκB phosphatase when cells are stimulated by external factors. Subsequently, the activated IκB‐α is ubiquitinated and degraded by proteasome [38, 39]. IκB‐α translocation between the cytoplasm and nucleus is mainly attributed to its special domains (i.e. NLS and NES) and the assistance of CRM‐1. As a compensation for excessive extranuclear IκB‐α, the p65 subunit continuously promotes gene transcription in the nucleus [40, 41].

Previous studies by our group have revealed that Y219 blocked the phosphorylation of IκB‐α, thus attenuating the activity of NF‐κB [20]. Besides, the combination therapy restrained the expression levels of downstream effectors of p65, which are associated with tumor growth, metastasis, and angiogenesis [42, 43, 44]. To confirm the conclusion drawn from *in vitro* data, the MDA‐MB‐231 xenograft model was then utilized to investigate the *in vivo* efficacy of the combination therapy. As a result, it has been verified that the combination therapy significantly suppressed the tumor growth, reduced the expression of Ki‐67, and increased TUNEL‐positive cells, indicating that the reduction of tumor growth was the result of apoptosis.

In conclusion, our results demonstrated that the combination treatment of KPT‐330 and Y219 exerted synergistic antitumor effects by inhibiting the NF‐κB signaling pathway in TNBC cell lines. The results indicate that a future combination of KPT‐330 and Y219 holds great promise for the treatment of TNBC.

## Conflict of interest

The authors declare no conflicts of interest.

## Author contributions

EB, ML, YZhu, and JW contributed to the study conception and design. EB, XW, HM, ZC, HZ, and JS acquired the data. MG, EB, and TW analyzed and interpreted the data. TW and YZhang wrote or revised the article. All authors read and approved the article.

### Peer review

The peer review history for this article is available at https://publons.com/publon/10.1002/2211‐5463.13588.

## Supporting information


**Fig. S1.** Combination of KPT‐330 and Y219 triggers G2‐M cell cycle arrest and apoptosis. (A‐C) The protein expression levels of cell cycle‐related proteins (p53, p21CIP1/WAF1, p27KIP1 and CCNB1) and apoptosis‐related proteins (cleaved caspase 3/8/9, PARP, BCL‐2, BAX and Cyto‐c) were determined by western blot in MDA‐MB‐231 cells treated with KPT‐330 (100 nm) and/or Y219 (6 nm) for 48 h. Actin was used as a control.

**Fig. S2.** KPT‐330 and Y219 synergistically inhibits NF‐κB activity. (A) The protein levels of NF‐κB related proteins (p65, IκB‐α, MMP‐2, VEGF and IL‐8) were detected by western blot in MDA‐MB‐231 cells treated with Y219 (6 nm) and/or KPT‐330 (100 nm) for 48 h. Actin was used as a control. (B‐C) Nuclear and cytoplasmic proteins of CRM1, Survivin, p65 and IκB‐α were determined by western blot in MDA‐MB‐231 cells treated with Y219 (6 nm) and/or KPT‐330 (100 nm) for 48 h. GAPDH, Actin, Brg1 and Lamin B1 serve as the internal controls.

**Table S1.** Details of the oligonucleotides used in this study.
**Table S2.** The IC_50_ of KPT‐330 and Y219 in different cell lines.
**Table S3.** The data of the combination index (Fig. 1D).

## Data Availability

The data that support the findings of this study are available in the figures and the supporting information.
