# Supplementary figures and images for: KPT‐330 and Y219 exert a synergistic antitumor effect in triple‐negative breast cancer through inhibiting NF‐κB signaling
Source: FEBS Open Bio. 2023 Mar 20;13(4):751–62. doi: 10.1002/2211-5463.13588 (PMC10068319; doi:10.1002/2211-5463.13588)

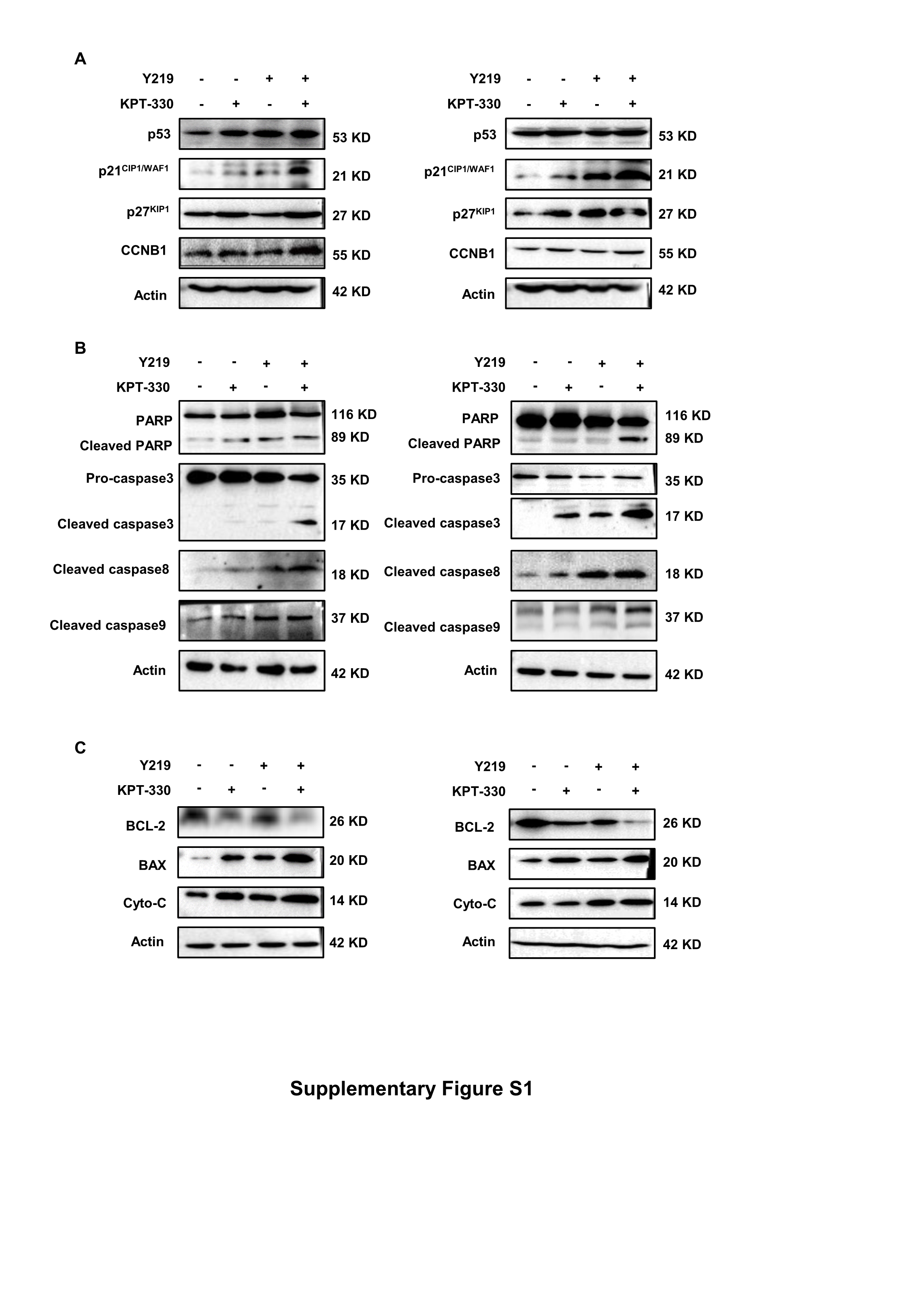

Supplement: Supplementary file 1 — Fig. S1. Combination of KPT‐330 and Y219 triggers G2‐M cell cycle arrest and apoptosis. (A‐C) The protein expression levels of cell cycle‐related proteins (p53, p21CIP1/WAF1, p27KIP1 and CCNB1) and apoptosis‐related proteins (cleaved caspase 3/8/9, PARP, BCL‐2, BAX and Cyto‐c) were determined by western blot in MDA‐MB‐231 cells treated with KPT‐330 (100 nm) and/or Y219 (6 nm) for 48 h. Actin was used as a control. [file FEB4-13-751-s003.jpeg]

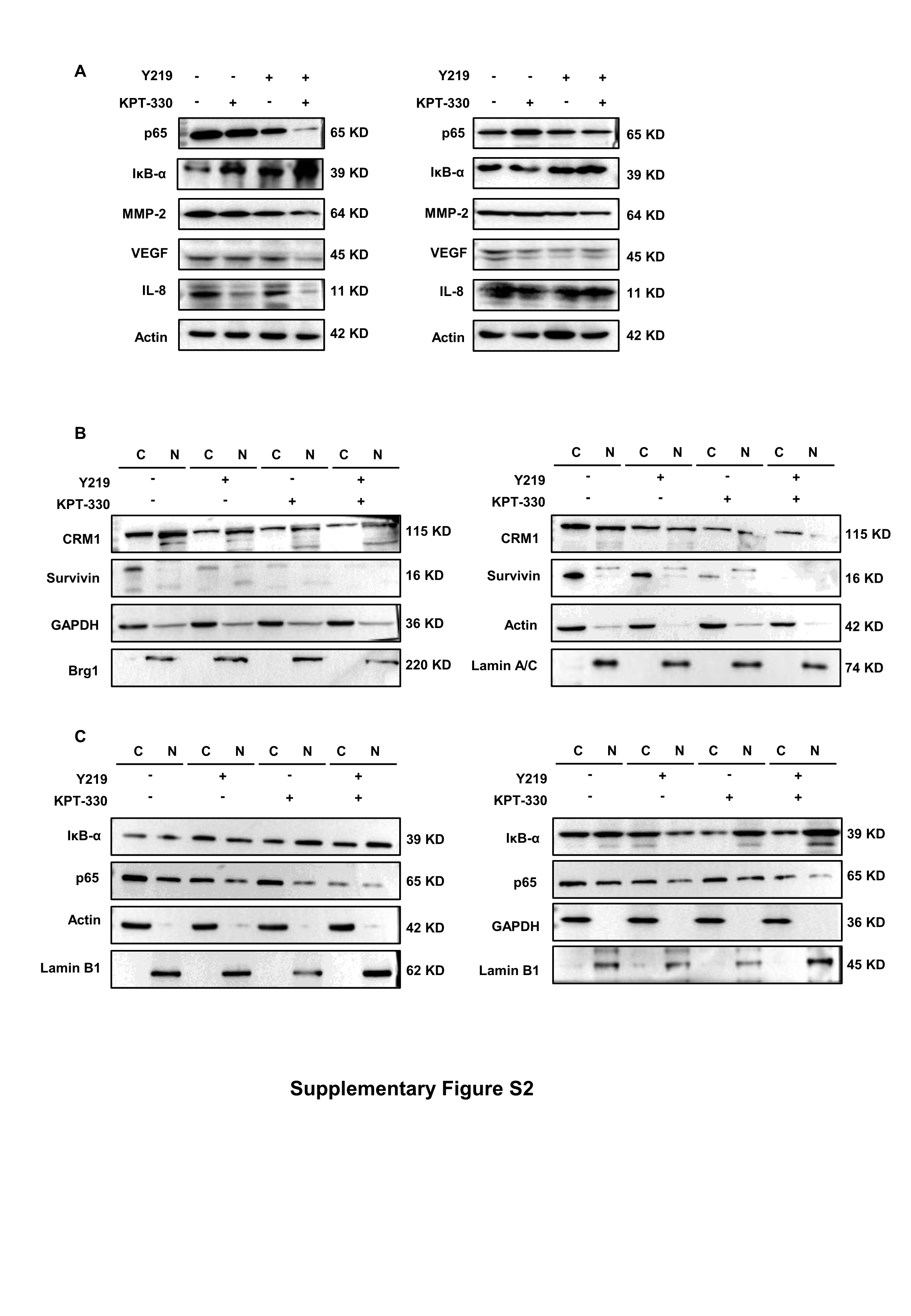

Supplement: Supplementary file 2 — Fig. S2. KPT‐330 and Y219 synergistically inhibits NF‐κB activity. (A) The protein levels of NF‐κB related proteins (p65, IκB‐α, MMP‐2, VEGF and IL‐8) were detected by western blot in MDA‐MB‐231 cells treated with Y219 (6 nm) and/or KPT‐330 (100 nm) for 48 h. Actin was used as a control. (B‐C) Nuclear and cytoplasmic proteins of CRM1, Survivin, p65 and IκB‐α were determined by western blot in MDA‐MB‐231 cells treated with Y219 (6 nm) and/or KPT‐330 (100 nm) for 48 h. GAPDH, Actin, Brg1 and Lamin B1 serve as the internal controls. [file FEB4-13-751-s002.jpeg]
